# Immunogenicity of the Two mRNA SARS-CoV-2 Vaccines in a Large Cohort of Dialysis Patients

**DOI:** 10.3390/idr14060093

**Published:** 2022-11-24

**Authors:** Paraskevi Tsoutsoura, Efstathios Xagas, Kyriaki Kolovou, Polyxeni Gourzi, Sotirios Roussos, Angelos Hatzakis, Ioannis N. Boletis, Smaragdi Marinaki

**Affiliations:** 1 Clinic of Nephrology and Renal Transplantation, Laiko General Hospital, Medical School of Athens, National and Kapodistrian University, 11527 Athens, Greece; 2 Molecular Immunopathology and Histocompatibility Unit, Onassis Cardiac Surgery Center, 17674 Athens, Greece; 3 Department of Hygiene, Epidemiology & Medical Statistics, School of Medicine, National and Kapodistrian University of Athens, 15772 Athens, Greece

**Keywords:** SARS-CoV-2 vaccination, hemodialysis, mRNA vaccine

## Abstract

Chronic kidney disease patients, especially those on hemodialysis, are at the highest risk of a severe course and death from COVID-19. Moreover, they appear to have suboptimal response in both cellular and humoral immunity after vaccination. The present study investigated humoral and cellular response and safety after two doses of either of the two authorized mRNA vaccines in a cohort of 310 patients on maintenance dialysis. The antibody response rate was 94.5%, with a median (25th, 75th) antibody titer of 3478 (1236, 8141) AU/mL. Only mild adverse effects were observed. Only vaccine type was independently associated with immunogenicity. Α statistically significant difference in favor of mRNA1273 versus BNT162b2 vaccine was observed. Antibody positivity (100% vs. 94.3%, *p* < 0.001), median (25th, 75th) antibody levels: 9499 (6118, 20,780) AU/mL vs. 3269 (1220, 7807) AU/mL (*p* < 0.001). Among the 65 patients tested for T-cell response, 27 (41.5%) had a positive one with a median (25th, 75th) antibody titer of 6007 (3405, 12,068) AU/mL, while 38 with no T-cell response presented a lower median (25th, 75th) antibody titer of 1744 (850, 4176) AU/mL (*p* < 0.001). Both mRNA vaccines are safe for dialysis patients and can trigger humoral and cellular responses, although with lower titers than those that have been reported to healthy individuals.

## 1. Introduction

More than 600 million confirmed cases of COVID-19, including 6,320,000 deaths, have been reported to the WHO (World Health Organization) until September 2022, while a total of 12,613,484,608 vaccine doses have been administered [1].

Patients with chronic kidney disease, especially kidney transplant recipients and patients on hemodialysis, are at the highest risk for a severe course and death from COVID-19 [2,3,4].

Although these patients are prioritized for COVID-19 vaccination in most countries, both groups were excluded from studies of the SARS-CoV-2 mRNA vaccines [5,6,7].

In addition, increasing evidence suggests lower response rates of these patients to both mRNA vaccines. In a recently published systematic review, the pooled estimated antibody response after complete vaccination in dialysis patients was 89% [8].

In another systematic review, hemodialysis patients’ proportions of humoral and cellular immune responses varied from 87.29% (80.77–93.81) to 88.78% (86.76–90.80) and 62.86% (56.56–69.17) to 85.78% (78.99–92.57), respectively, between the first and fourth week [9].

The suboptimal response in both cellular and humoral immunity in this population is not surprising. Patients on maintenance dialysis have a reduced response to vaccination to hepatitis B due to uremia-associated suppression of the immune system with disturbances in T-lymphocytes and antigen-presenting cells, which result in lower protective antibody titers and an inability to maintain adequate antibody titers over time [10,11].

Additionally, available data show that COVID-19 vaccines may be less effective in immunocompromised populations. A systematic review was published recently that assessed immunogenicity, efficacy, and effectiveness of COVID-19 vaccines in various immunocompromised populations, which included 157 studies with a total of 25,209 participants, of whom 6302 were on dialysis (25.0%). Proportion of non-responders in the dialysis group ranged from 2 to 30%, which was lower than in solid organ transplant recipients (range 18–100%) and in patients with hematological malignancy (range 14–61%) [12].

As a result, additional data on the efficiency of SARS-CoV-2 vaccination are needed not only to understand the protection vaccination will or will not afford patients with ESRD (End Stage Renal Disease) but also to assess whether a re-evaluation of vaccination strategies regarding the population of patients on hemodialysis is needed.

In the present study, we aimed to assess humoral and cellular immunity response as well as safety after full vaccination with one of the two mRNA SARS-COV-2 vaccines in a large cohort of 310 patients on maintenance dialysis. Furthermore, we investigated differences in response rates between the BNT162b2 (Pfizer, New York, NY, USA/BioNTech, Mainz, Germany) and the mRNA1273 (Moderna, Cambridge, MA, USA) vaccine.

## 2. Materials and Methods

We included a total of 310 patients on maintenance hemodialysis vaccinated with either of the two authorized mRNA SARS-COV-2 vaccines. All patients had completed their vaccination with either BNT162b2 or mRNA1273 and were scheduled for a blood specimen 3 to 4 weeks after the second vaccine dose in order to determine their humoral and cellular response. Participants were asked to fill out a questionnaire including demographics, previous COVID-19 infection, and early post-vaccination side effects after each vaccine dose. Previous COVID-19 infection was not an exclusion criterion since, according to national vaccination policy, patients recovering from natural infection were advised to proceed to a full vaccination two months later. However, according to medical history, none of the participants has a known COVID-19 infection before antibody measurement.

All laboratory tests were performed in Onassis Cardiac Surgery Center and were subsequently recorded in the patients’ charts.

Antibodies were measured at a median of 29 days after the 2nd vaccine dose (range, 27 to 30 days) utilizing a chemiluminescent microparticle immune assay (CMIA) which quantifies IgG antibodies against the receptor-binding domain (RBD) of the S1 subunit of the spike protein of SARS-CoV-2 (Abbott SARS-CoV-2 IgG II Quant). The linear range of the assay is between 21 and 40,000 Arbitrary Units per milliliter (AU/mL) and the lower limit of detection (LoD) 6.8 AU/mL. The clinical specificity is estimated at 99.55% (95% confidence interval (CI), 99.15–99.76%) and the clinical sensitivity at 98.81% (95% CI 93.56–99.94%) in samples collected ≥15 days after a positive PCR (polymerase chain reaction) at a cut-off value of 50 AU/mL, according to the manufacturer’s suggestions. Anti-SARS- CoV-2 RBD IgG assays have shown an excellent correlation with neutralizing antibodies [13,14].

Cellular response was available in a subset of the study participants, which were randomly selected. In the matter of cellular immunogenicity, we used the interferon gamma (IFN-γ) release assay (Qiagen, Hilden, Germany) (IGRA), QuantiFERON SARS-CoV-2, which is an in vitro blood diagnostic to measure IFN-γ released by antigen-specific T cells after overnight stimulation with pathogen-specific peptides. A whole blood IGRA was used for the simple and high-throughput detection of cellular immune response to SARS-CoV-2.

### Statistical Analysis

Median values, 25th and 75th percentiles were used to describe anti-RBD SARS-CoV-2 antibody levels. Samples below LoD 6.8 AU/mL were assigned the value 6.8 AU/mL. The non-parametric Mann–Whitney U test and Fisher’s exact test were used to compare antibody levels and vaccine type. A Poisson regression model with robust standard error was used to identify factors associated with immune response. We conducted all statistical analyses using Stata 13.1 (StataCorp, College Station, TX, USA).

## 3. Results

We recruited a total of 310 patients on maintenance hemodialysis from 8 hemodialysis centers of Athens. Of the total 310 patients, 204 (60.8%) were male. The mean (SD) age was 63.1 (13.3) years old. Of the 310 patients, 262 (84.5%) were born in Greece, while 48 (15.5%) were of a different ethnicity. The median (25th, 75th) age at the start of maintenance hemodialysis was 61.4 (49.6, 68.7). On study inclusion, the median (25th, 75th) time between the start of hemodialysis and 2nd dose was 3.8 (1.5, 6.6) years (Table 1).

### 3.1. Safety

The vast majority of patients (95.8%) had received the BNT162b2 and the remaining (4.2%) the mRNA1273 vaccine. Overall, both vaccines were well tolerated. As presented in Table 2, no side effects after the 1st injection reported 120 (38.7%) patients, while pain at the injection site was the most commonly reported local reaction, occurring in 133 (42.9%) of all patients. Other reported side effects were fatigue (10.7%), headache (8.1%), muscle pain (6.5%), fever (2.3%) and skin rash (0.3%).

After the second injection, 137 (29.2%) patients reported pain at the injection site, while 34 (11%) of them complained about headache. The occurrence of fatigue, muscle pain and fever increased after the second injection, affecting 48 (15.5%), 37 (11.9%) and 28 (9%), respectively. An overview of all local and systemic reactions after the vaccination is given in Table 2.

### 3.2. Humoral Response

We found that 293 (94.5%) of the total of 310 patients tested positive for anti-SARS-COV-2 RBD-IgG antibodies, with a median (25th, 75th) antibody titer of 3478 (1236, 8141) AU/mL (Table 3).

Specifically, 280 (94.3%) of the 297 patients who were vaccinated with BNT162b2 vaccine tested positive, with a median (25th, 75th) antibody titer of 3269 (1220, 7807) AU/mL while all (100%) of the 13 patients who were vaccinated with mRNA-1273 were tested positive, with median (25th, 75th) antibody titer of a 9499 (6118, 20,780) AU/mL (Supplementary Data S1).

### 3.3. Comparison of Response Rate between the Two mRNA Vaccines

We found a statistically significant difference in favor of the mRNA1273 versus BNT162b2 vaccine in terms of antibody positivity (100% vs. 94.3%, *p* < 0.001), as well as median (25th, 75th) antibody levels: 9499 (6118, 20,780) AU/mL compared to 3269 (1220, 7807) AU/mL (*p* < 0.001), respectively (Table 3).

Finally, univariable and multivariable analyses were performed to identify factors associated with immune response. From the covariates assessed, only vaccine type (Moderna vs. Pfizer/BioNTech) was independently associated with immunogenicity (*p* < 0.001) (Table 4) (Supplementary Data S2).

### 3.4. Cellular Response

We randomly selected 65 of the total cohort of 310 patients to test cellular response to SARS-COV-2. Of these, 63 had tested positive for anti-SARS-COV-2 RBD-IgG antibodies, while 2 of them tested negative. We observed a positive specific T-cell response for SARS-CoV in 27 (42.9%) of those who had a positive antibody response, while 36 of them were not responsive (57.1%) (*p* = 0.507).

As shown in Table 5, those 27 patients with a positive specific T-cell response presented a median (25th, 75th) antibody titer of 6007 (3405, 12,068) AU/mL, while those 38 patients with no T-cell response presented a lower median (25th,75th) antibody titer of 1744 (850, 4176) AU/mL (*p* < 0.001) (Table 5).

## 4. Discussion

Patients with chronic kidney disease are at high risk for a severe course and death from COVID-19, especially patients in maintenance dialysis who cannot effectively self-isolate and reduce contacts to avoid infection due to their regular need for therapy [2,3,4].

However, this cohort of patients was excluded from the initial studies of the SARS-CoV-2 mRNA vaccines. As a result, it is of great importance to study the safety and efficacy of the vaccines in order to gain insight on the achieved immunogenicity and reassess and modify each country’s vaccination strategies worldwide [5,6,7,15].

The present study is, to the best of our knowledge, one of the largest studies investigating antibody response rates and safety of the two mRNA SARS-COV-2 vaccines in hemodialysis patients. Most importantly, we compared the immunogenicity of the two available mRNA vaccines, as well as cellular and humoral response, and we provide data about clinical effectiveness which are urgently needed.

We showed that response rates were at 94.5% among hemodialysis patients approximately 4 weeks after complete vaccination, which was similar to the response rate of 88.78% described in a recently published systematic review including 27 studies with a total of 1337 patients on hemodialysis [9].

Schrezenmeier et al., in a small observational study included 36 patients on dialysis vaccinated with two doses of Pfizer BNT162B2. Humoral immunogenicity was assessed by SARS-CoV-2 specific IgG and IgA antibodies, and specific T-cell responses were assessed by an interferon-gamma release assay (IGRA), while outcomes were compared to a group of 44 elderly patients not on dialysis. Only 32/36 patients (88.9%) demonstrated IgG detection, and compared to a cohort of vaccinees with similar age but not on chronic dialysis, seroconversion rates and antibody titers were significantly lower [16].

Broseta et al., in one of the largest studies including 205 patients treated at three dialysis units in Barcelona and vaccinated with either the mRNA-1273 or BNT162b2 vaccine, found that 97.7% of 175 vaccinated patients who were seronegative at baseline developed a response (humoral, cellular, or both); 95.4% of these patients seroconverted [17].

Regarding cellular immunogenicity, we observed a positive specific T-cell response in only 27 (42.9%) out of 63 patients with a positive antibody response (*p* = 0.507). However, there was no statistically significant correlation between the two immune responses (*p* = 0.507). This response was lower than what Schrezenmeier et al., reported, specifically for 21/31 vaccinated dialysis patients (67.7%) compared to 42/44 (93.3%) in controls of similar age [16]. Similarly, Broseta et al. reported 62% of those tested for cellular immunity having a positive response [17].

However, the frequency distribution of antibody levels by cellular immune response showed that 27 (41.5%) of our 65 patients that had a positive cellular response presented a median (25th, 75th) antibody titer of 6007 (3405, 12,068) (AU/mL), in comparison with the 38 (58.5%) with no cellular response who had a median (25th, 75th) antibody titer of 1744 (850, 4176) (AU/mL). Positive cellular response significantly correlated with higher levels of anti–S1-RBD IgG (*p* < 0.001).

We also investigated the factors associated with positive antibody titer. We did not find any statistically significant correlation between age, gender, time from the start of hemodialysis or age at the start of hemodialysis and antibody production. The only statistically significant correlation was between antibody titer and the Moderna vaccine with RR (95% CI) 1.06 (1.03–1.09) (*p* < 0.001).

Danthu et al. presented that the humoral response to the Pfizer BNT162B2 vaccine in patients undergoing hemodialysis is guided by factors related to the uremic condition, leading to delayed humoral response of lower magnitude, while according to Broseta et al., a greater age and immunosuppressive treatment were associated with lower antibody levels [17,18].

It is well-established that patients on dialysis have major reasons for low humoral response to vaccination. Factors such as malnutrition, anemia, high PTH values, and lower solute clearance, as measured by urea Kt/V, have been correlated with this observation. Possible pathogenetic mechanisms include dysregulated cytokine synthesis, abnormalities in T-lymphocytes with decreased lymphocyte proliferation, lymphopenia and alterations in B-lymphocyte function, which results in decreased immunoglobulin production [10,11].

A head-to-head comparison of the two mRNA vaccines revealed a difference in favor of the mRNA1273 vaccine in terms of creating a more robust humoral immunity regarding response rates and antibody titers as well. Of a total of 297 patients who were vaccinated with the BNT162b2 vaccine, 280 tested positive (94.3%), while all 13 patients who were vaccinated with mRNA-1273 (100%) tested positive. The mRNA-1273 positively significantly correlated with higher levels of antibodies (*p* < 0.001).

A similar conclusion was reached by Ionita et al., who recently published a large study including 398 patients on maintenance hemodialysis, 303 of whom received the BNT162b2 and 95 received the mRNA-1273 vaccine. The adjusted OR of developing a sufficient antibody level between the two vaccines was 3.91 (*p* = 0.077) in favor of mRNA-1273. While both of the mRNA-based vaccines showed good efficacy, those preliminary data may suggest a higher antibody response to the mRNA-1273 vaccine [19].

These data are also compatible with a recently published study of our clinic, regarding immunogenicity in solid organ transplant recipients. We have recently showed that in 455 SOT recipients vaccinated with the mRNA1273 and BNT162b2 vaccine, the antibody response rate was low, at 39.6%. Higher immunogenicity was detected among individuals vaccinated with the mRNA1273 compared to those with the BNT162b2 vaccine (47% vs. 36%, respectively, *p* = 0.025) as well as higher median antibody levels of 31 (7, 372) (AU/mL) vs. 11 (7, 215) AU/mL, respectively [20].

Our findings in both the aforementioned and the present study are compatible with the findings Sanders et al. reported a few months ago in their multicenter study called RECOVAC. All participants have received two doses of the mRNA1273 vaccine. The response rates are 56.9% for kidney transplant recipients and 99.4% for dialysis patients [21].

Regarding safety, data on adverse events after vaccination with both the mRNA vaccines in hemodialysis patients is reassuring [20]. In accordance with previous studies, we recorded only mild reactions in about two-thirds of our patients, with predominantly local pain occurring after both doses. Interestingly, even though a number of patients are on anticoagulants, no bleeding events have been reported in our cohort. The frequency of vaccine side effects appears to be lower than in the general population. It is not known if the immune response dysregulation in HD patients has also an influence on the manifestation of AEs [8,10]. Further studies are needed to reveal a potential relationship between the occurrence of AEs and the immune response of dialysis patients.

Apart from the difference in seroconversion between the two mRNA vaccines, our main finding remains the reassuring high immune response of dialysis patients. Their response appears to be much better than in kidney transplant recipients, but it remains inferior to response in general population.

Strengths of our study are that it includes one of the largest cohorts of hemodialysis patients in whom mRNA vaccine immunogenicity has been investigated until today, including patients from several large dialysis units of the Athens metropolitan area. Novelties of our study are the head-to-head comparison of the two mRNA vaccines and correlation of humoral immunity with antibodies and cellular immunity.

Limitations include the absence of a healthy control group and the lack of testing of antibodies to nucleocapsid protein, which along with patient history, it would ensure distinction between previously infected and naïve-vaccinated patients. In addition, there was a lack of information about patient’s follow-up and any breakthrough infection post vaccination.

## 5. Conclusions

In conclusion, our study’s results are compatible with the contemporary studies worldwide, suggesting that both mRNA vaccines are safe for patients in maintenance hemodialysis and can trigger a weaker humoral immunogenicity than in healthy subjects. Furthermore, we studied cellular immunogenicity and showed a weaker SARS-COV-2 specific T-cell response than other studies; however, our correlations were statistically non-significant. Our findings will add to the understanding of vaccine immunogenicity and reassessment of the vaccination policy regarding this immunocompromised patient population.

## Figures and Tables

**Table 1:** Demographic characteristics of study population.

	*n* = 310
Gender, *n* (%)	
Male	204 (65.8)
Female	106 (34.2)
Age at the 1st vaccine dose, mean (SD) ^1^	63.1 (13.3)
Age at the 1st vaccine dose, median (25th, 75th) ^2^	66.7 (55.0, 72.0)
Country of birth, *n* (%)	
Greece	262 (84.5)
Other	48 (15.5)
Age at the start of dialysis, mean (SD) ^1^	57.4 (15.5)
Age at the start of dialysis, median (25th, 75th) ^2^	61.4 (49.6, 68.7)
Time period between the start of dialysis and 2nd dose (years), median (25th, 75th) ^2^	3.8 (1.5, 6.6)
Time period between the start of dialysis and 2nd dose (years), *n* (%)	
<1	53 (17.1)
[1–3)	71 (22.9)
[3–6)	97 (31.3)
≥6	89 (28.7)

^1^ Standard deviation, ^2^ 25th, 75th percentile.

**Table 2:** Vaccine type and side effects after vaccination among dialysis patients, *n* = 310.

	*n* = 310
Vaccine Type, *n* (%)	
Pfizer–BioNTech	297 (95.8)
Moderna	13 (4.2)
Side effects after the first dose of COVID-19 vaccine, *n* (%)	
Yes	190 (61.3)
No	120 (38.7)
Side effects after the first dose of COVID-19 vaccine ^1^, *n* (%)	
Pain on the arm	133 (42.9)
Headache	25 (8.1)
Tiredness	33 (10.7)
Muscle pain	20 (6.5)
Fever	7 (2.3)
Skin rash	1 (0.3)
Other	6 (1.9)
Side effects after the second dose of COVID-19 vaccine, *n* (%)	
Yes	198 (63.9)
No	112 (36.1)
Side effects after the second dose of COVID-19 vaccine ^1^, *n* (%)	
Pain on the arm	137 (44.2)
Headache	34 (11.0)
Tiredness	48 (15.5)
Muscle pain	37 (11.9)
Fever	28 (9.0)
Skin rash	4 (1.3)
Other	12 (3.9)

^1^ Some participants had more than one symptom.

**Table 3:** Antibodies levels in hemodialysis patients by vaccine (BNT162b2 vs. mRNA-1273).

	Total	BNT162b2	mRNA-1273	
	*n* = 310	*n* = 297	*n* = 13	*p*-Value
Antibodies (AU/mL), median (25th, 75th) ^1^	3478 (1236, 8141)	3269 (1220, 7807)	9499 (6118, 20,780)	0.001 ^2^
Antibody levels, *n* (%)				1.000 ^3^
Negative (<50 AU/mL)	17 (5.5)	17 (5.7)	0 (0.0)	
Positive (≥50 AU/mL)	293 (94.5)	280 (94.3)	13 (100.0)	

^1^ 25th, 75th percentile, ^2^ Mann–Whitney U test, ^3^ Fisher’s exact test.

**Table 4:** Factors associated with the incidence of positive IgG II among patients, *n* = 310.

Variable	Univariable RR (95% CI)	*p*-Value	Adjusted Multivariable RR (95% CI)	*p*-Value
Age (years)	1.00 (1.00–1.00)	0.730	1.00 (1.00–1.00)	0.768
Age at the start of dialysis (years)	1.00 (1.00–1.00)	0.554		
Time period between the start of dialysis and 2nd dose (years)	1.00 (1.00–1.00)	0.423		
Gender		0.922		0.940
Male	Ref.		Ref.	
Female	1.00 (0.94–1.06)		1.00 (0.94–1.06)	
Vaccine		<0.001		<0.001
Pfizer/Biontech	Ref.		Ref.	
Moderna	1.06 (1.03–1.09)		1.06 (1.03–1.09)	

Abbreviations: RR, risk ratio; CI, confidence interval.

**Table 5:** Frequency distribution of antibody levels by cellular immune responses in COVID-19 (*n* = 65).

	*n* (%)	Antibody Response Median (25th, 75th) ^1^	*p*-Value
Antibodies (AU/mL)	65 (100.0)	3457 (1186, 7146)	
Cellular immunity			<0.001 ^2^
No	38 (58.5)	1744 (850, 4176)	
Yes	27 (41.5)	6007 (3405, 12,068)	

^1^ 25th–75th percentile, ^2^ Mann–Whitney U test.

## Data Availability

The datasets used and/or analyzed during the current study are available from the corresponding author on reasonable request.

## Supplementary Materials

The following supporting information can be downloaded at: https://www.mdpi.com/article/10.3390/idr14060093/s1; Supplementary Data S1: Antibody-negative cases, Supplementary Data S2: Multivariable analysis assessing factors associated to antibody levels after the full vaccination in dialysis patients [22,23].
