# Supplementary material for: Immunogenicity of the Two mRNA SARS-CoV-2 Vaccines in a Large Cohort of Dialysis Patients
Source: Infect Dis Rep. 2022 Nov 24;14(6):946–54. doi: 10.3390/idr14060093 (PMC9778780; doi:10.3390/idr14060093)
Supplement: Supplementary file 1 [file idr-14-00093-s001.zip › Supplementary Data S2.pdf]

The antibody levels were transformed on a Log10 scale, to normalize their distribution [1, 2]. A linear regression model was used to assess associations between log-transformed IgG antibody titers and the following variables: age, gender, vaccine type, side effects after 1st dose of vaccine, side effects after 2nd dose of vaccine, age when hemodialysis started, and time period from dialysis to the 2nd dose of vaccine.

Supplementary Table 1. Multivariable analysis assessing factors associated to antibody levels after the full vaccination in dialysis patients, N = 310.

**Variable**	**Beta coefficient (SE)**	**p-value**
Age (years)	-0.008 (0.004)	0.020
Gender		
Male	Ref.	
Female	-0.123 (0.096)	0.202
Vaccine		
Pfizer/Biontech	Ref.	
Moderna	0.469 (0.227)	0.040
Side effects after the second dose of COVID-19 vaccine		
No	Ref.	
Yes	0.242 (0.100)	0.016

1. Rus, K.R., et al., *Performance of the rapid high-throughput automated electrochemiluminescence immunoassay targeting total antibodies to the SARS-CoV-2 spike protein receptor binding domain in comparison to the neutralization assay.* Journal of Clinical Virology, 2021. **139**: p. 104820.
2. Sormani, M.P., et al., *Effect of SARS-CoV-2 mRNA vaccination in MS patients treated with disease modifying therapies.* EBioMedicine, 2021. **72**: p. 103581.
